# Adherence to the nordic diet is associated with anxiety, stress, and depression in recovered COVID-19 patients, a case-control study

**DOI:** 10.1186/s40795-024-00845-x

**Published:** 2024-03-01

**Authors:** Asie Araste, Mohammad Reza Shadmand Foumani Moghadam, Kimia Mohammadhasani, Mohammad Vahedi Fard, Zahra Khorasanchi, MohammadReza Latifi, Elahe Hasanzadeh, Nasrin Talkhi, Payam Sharifan, Parisa Asadiyan-Sohan, Marjan Khayati Bidokhti, Arezoo Ghassemi, Reza Assaran Darban, Gordon Ferns, Majid Ghayour-Mobarhan

**Affiliations:** 1 Department of Nutrition, School of Medicine, Mashhad University of Medical Sciences, Mashhad, Iran; 2 Student Research Committee, faculty of Medicine, Mashhad University of Medical Sciences, Mashhad, Iran; 3 BSc. in Nutrition Sciences, Varastegan Institute for Medical Sciences, Mashhad, Iran; 4 Department of Nutrition, Food Sciences and Clinical Biochemistry, School of Medicine, Social Determinants of Health Research Center, Gonabad University of Medical Science, Gonabad, Iran; 5 International UNESCO Center for Health Related Basic Sciences and Human Nutrition, Mashhad University of Medical Sciences, Mashhad, Iran; 6 Department of Biostatistics, School of Health, Mashhad University of Medical Sciences, Mashhad, Iran; 7 Departments of Biology, Faculty of Sciences, Mashhad Branch, Islamic Azad University, Mashhad, Iran; 8 Brighton and Sussex Medical School, Division of Medical Education, Brighton, UK

**Keywords:** Nordic diet, COVID-19, Whole grain, Fruit, Anxiety, Stress, Depression

## Abstract

**Background:**

Follow-up of COVID-19 recovered patients to discover important adverse effects on other organs is required. The psychological health of COVID-19 patients may be affected after recovery.

**Aim:**

We aimed to evaluate the association between adherence to the Nordic diet (ND) and psychological symptoms caused by COVID-19 after recovery.

**Method:**

Dietary data on 246 qualified adults (123 cases and 123 controls). The dietary intake in this case-control study was calculated by a reliable and valid food frequency questionnaire (FFQ). Depression Anxiety Stress Scale (DASS), Pittsburgh Sleep Quality Index (PSQI), Insomnia Severity Index (ISI), and Short-Form Health Survey (SF-36) were used to analyze participant’s anxiety, stress, depression, sleep quality, insomnia, and quality of life of participants.

**Results:**

There was a significant inverse relationship between total anxiety, stress, and depression scores and the intake of whole grains (*P* < 0.05). Furthermore, there was a significant inverse association between depression and fruit intake (*P* < 0.05). A significant negative correlation was found between insomnia and sleep quality and the intake of root vegetables (*P* < 0.05). In the multinomial-regression model, a significant association between the Nordic diet and anxiety, stress, and depression was found only in the case group (OR = 0.719, 95% CI 0.563–0.918, p-value = 0.008; OR = 0.755, 95% CI 0.609–0.934, P-value = 0.010, and, OR = 0.759, 95% CI 0.602–0.956, P-value = 0.019 respectively).

**Conclusion:**

Adherence to the Nordic diet might reduce anxiety, stress, and depression in recovered COVID-19 patients.

## Introduction

Coronavirus disease 2019 (COVID-19) is an infectious disease that caused patients a wide range of physical and psychological problems [1]. Some studies noticed COVID-19 patients experience psychological and psychiatric problems after infection such as insomnia, anxiety, depression, delirium, memory loss, and loss of concentration [2, 3]. These psychological symptoms may continue after convalescence from COVID-19 and harm the mental health of recovered patients. Impaired mental health reduces the quality of life [4], so it should be considered during hospitalization and recovery. These patients suffer from psychological sequelae after COVID-19, so it is essential to follow up patients that recovered from COVID-19 [5]. Li et al. reported that 35% of COVID-19 patients have severe to moderate psychological symptoms [6]. A systematic review found anxiety and insomnia to occurred in 35.7% and 41.9% of patients with acute SARS, falling to 12.3% and 12.1% at follow-up [4].

Dietary interventions with relatively moderate effect sizes can significantly reduce the mental and neurological disease burden through food and nutrient-based approaches [7, 8]. ND is a “plant-based” dietary pattern that recommends protein intake from plant sources. It also recommends consumption of fruits and vegetables, whole grains, seeds, and nuts [9]. To increase protein intake, ND recommends increased the consumption of legumes and fish [9]. Along with all these recommendations, there is a limit on the use of red meat and processed foods [10]. Some studies have shown that ND has beneficial effects on psychological symptoms [10, 11]. Therefore, it is essential to assess the diet of COVID − 19 recovered patients based on their symptoms. Nutraceutical interventions are increasingly being used in psychiatric practice [5]. Jacka et al. demonstrated impressive effects of a 3-month dietary intervention on moderate-to-severe depression with a 32% remission in the intervention group [12]. Another study of the Nordic diet reported better improvement in depression in the ND group compared to the control group [13].

Recovered COVID-19 patients faced a severely stressful experience that challenged their psychological health. It is necessary to follow up these people and control their diet to improve their impaired mental health. Considering a healthy diet can improve psychological symptoms after COVID-19. Few studies have been performed to analyse the Nordic diet on psychological disorders. In this case-control study, we attempted to investigate the association between adherence to the ND and psychological responsibility in both recovered COVID-19 patients and healthy people.

## Method

### Study design

The present case-control study was performed between November 2020 to January 2021 at the clinic of Qaem Hospital, Mashhad, Iran. Adult subjects aged ≥ 30 years old who were affected by COVID-19 within the last 1 month. Participants had a negative CT scan or PCR test for COVID-19 when the interview was started. Also, we randomly selected the control group from adults > 30 years who did not have a COVID-19 history. These participants were referred to the nutrition clinic of the Qaem Hospital. Subjects who had a history of depression treatment in the last 6 months, autoimmune diseases, cancer, renal or hepatic failure, and metabolic bone disease were excluded. Adherence to a special dietary pattern such as a vegetarian diet was another exclusion criteria.

In the control group, subjects with a history of COVID-19 according to CT scan or PCR test, renal or hepatic failure, autoimmune diseases, having a history of depression treatment in the last 6 months, cancer, and adherence to a special diet were excluded. We enrolled one matched control subject for every case. Also, the case and control groups corresponded according to age and gender (± 5 years). In the present study, 246 subjects who had the eligibility criteria were recruited, of which a total of 240 subjects (120 cases and 120 controls) were encompassed in the last analysis. The mean energy of two cases and four controls were outside ± 3 standard deviation (SD), so they were excluded. All cases and control filled out noticed written agreement, and all methods were performed based on related guidelines and regulations or the Helsinki affirmation.

### General and anthropometric characteristics

Demographic and anthropometric features, such as age, gender, height, weight, and education level were carried out by an expert nurse. Weight was measured by a calibrated personal scale. the fixed measuring tape was used to find out the height. Body weight (kg)/ (body height (m))^2^ was applied for calculating body mass index (BMI).

### Dietary intake assessment

The food intake of patients was determined by a reliable and valid 68-item semi-quantitative food frequency questionnaire (FFQ) [14, 15]. The FFQ was completed through face-to-face interviews. Food analysis was undertaken using Nutritionist IV software (N-Squared Computing, Cincinnati, OH, USA). Healthy Nordic Food Index (HNFI) scores were assessed based on the method of Olsen et al. [16]. To calculate the HNFI, we consider six groups with the same micronutrient amount. Daneshzad et al. [17] validated the modified ND score for the Iranian population, including (a) fish (fish conserved in oil and salt and other fish), (b) legumes (soybeans, beans, and lentils), (c) whole grains, (d) fruit (fresh and dried fruits, fruit juice) (e) root vegetables (onion, garlic, and potato) and (f) cabbages and vegetables (lettuce, tomato, cucumber, spinach, and leafy vegetables), We calculated below- and above-average intake for every item. Each group was classified based on the score obtained (scoring 0–1 points shows “low adherence”, scoring 2–3 points “medium adherence”, and scoring 4–6 points “high adherence”). ND was not given to any individuals and agreement to ND was assessed.

### Depression anxiety stress scales (DASS)

Depression anxiety stress scales (DASS) are among the most valid and exact tools to analyze mental conditions [18]. It is a questionnaire that generally includes three subscales, seven questions, and 21 items. Each question score ranges from 0 to 3 on a four-point scale to recognize the severity of mental disorders, consisting of depression, anxiety, and stress. In DASS, a lower score reveals a lower degree of negative mood, and a higher score indicates a more severe degree of negative emotion. In the Iranian population, the validity and reliability of the used version of DASS in this study, have been reported formerly [19]. The anxiety, stress, and depression scores were separated into two categories: No or minimal scores and some degree of mental disorder. According to the scores obtained from each item decided as follows: (≤ 7, No), (> 7, some degree of anxiety), (≤ 14, No), (> 14 some degree of stress), (≤ 9, No), (> 9, some degree of depression)

### Pittsburgh sleep quality index (PSQI)

The sleep quality of the patients was analyzed using a 19-item self-reported PSQI questionnaire that evaluates sleep quality during the last 30 days [20]. It consists of 19 objects combined for 7 component scores, containing sleep duration, sleep latency, subjective sleep quality, sleep disturbances, use of sleep medication, daytime dysfunction, and habitual sleep competence. The responses are scored on a 3-point scale, ranging from 0 to 3. The total score for sleep quality is measured by combining the 7 component scores, which range from 0 to 21. Subjects were categorized into two groups according to their PSQI score: the good-sleeper group (PSQI ≤ 5) and the poor-sleeper group (PSQI > 5). Also, the validity of the PSQI Persian version has been confirmed in 2012 [21].

### Insomnia severity index (ISI)

The Insomnia Severity Index (ISI) is a seven-item self-report tool for determining patients’ insomnia symptoms and their outcomes. The aspects measured included severeness of sleep onset, interference of sleep difficulties with daytime functioning, sleep dissatisfaction, early morning awakening problems, sleep preservation, distress caused by sleep difficulties, and noticeability of sleep problems by others [22]. According to severeness, each item scored on a 0–4 scale with a full scale ranging from 0 to 28. The scoring system reports as follows: severe insomnia [22–28], mild insomnia [15–21], sub-threshold insomnia [8–14], and no insomnia (0–7). In the Iranian population the reliability and validity of the Persian version of this questionnaire have been confirmed (Cronbach’s a > 0.8 and intra-class correlation coefficient > 0.7) [23].

### Quality of life questionnaire

We used the Short-Form Health Survey (SF-36) validated questionnaire to analyze the general quality of life. SF-36 calculated the overall healthy quality of life based on Mental Health, General Health, Vitality, Role Emotional, Social Functioning, Body Pain, Role Physical, and Physical Functioning. Scores of this questionnaire range from 0 to 100 and the higher score shows a higher quality of life. The SF-36 was assessed in the Iranian population in a prior study and revealed construct validity and good reliability [24].

### Statistical analysis

The Kolmogorov-Smirnov test was used to analyze the normality of variables. Descriptive statistics, such as SD and mean, were determined for all variables and expressed as mean ± SD for normally distributed variables and median and interquartile range (IQR) for non-normally distributed variables. Also, categorical indices were indicated by percent. We used Chi-square test and independent sample t-test to compare variables between case and control groups. For food intake comparison among two groups besides tertiles of HNFI score, a Multivariate Analysis of Variance (MANOVA) test was performed. Pearson correlation test was used to show an association between components of the Nordic diet and psychological scores. Eventually, we used multinomial logistic regression to evaluate the correlation between the classification of the adherence ND and psychological scores. Statistical package for social sciences (SPSS) version 18 (IBM Inc. Chicago, IL, USA) was used to perform statistical analyses, and rpart package in R version 4.1.2 (R Core Team. 2020). Statistical significance was considered as p-value < 0.05.

## Results

**Fig. 1:**
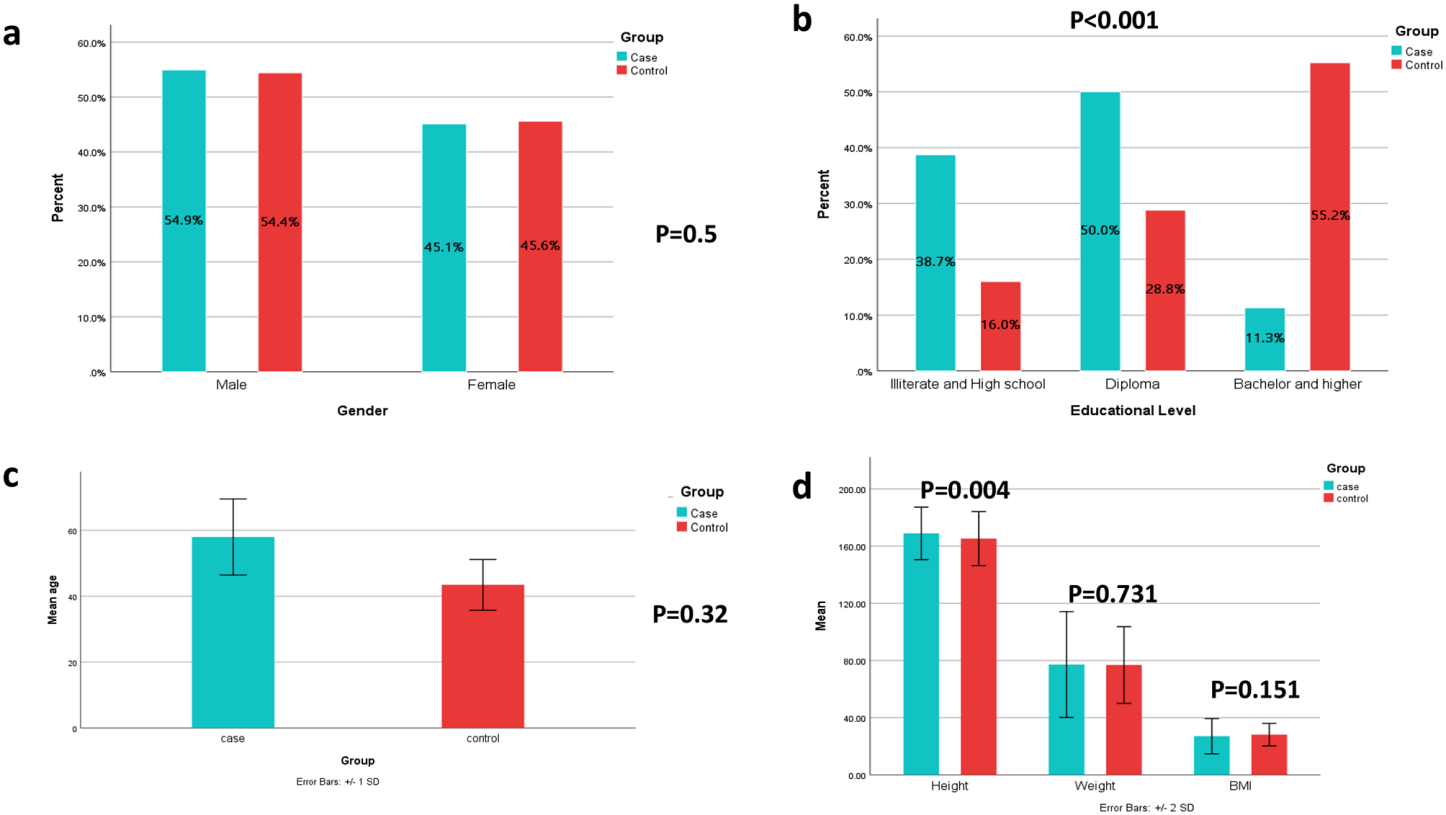
Demographic and clinical characteristics of the participants between groups. **(a)** Gender, **(b)** Education, **(c)** Age, **(d)** Anthropometric measurements

Demographic and anthropometric characteristics of the participants in case and control groups are shown in Fig. 1. The case and control group mean age was 60.38 ± 13.61 and 57.43 ± 7.71 years, respectively (Fig. 1c). The case group had 45% females and the control group had 45.6% females (Fig. 1a). There were no significant differences in gender, age, weight, and BMI between case and control groups (*p* > 0.05). Nevertheless, there was a significant difference in educational level and height between the two groups (*p* < 0.05) (Fig. 1b).

Table 1. demonstrates the comparison of the mean energy, macronutrients, and components of the HNFI score in classification of adherence ND between both groups. There was a significant difference in energy consumption between the case and control groups (*p* = 0.036). Regarding components of the HNFI score, there were significant differences between fruits, legumes, cabbage and vegetables, fish intakes, and classification of HNFI score in both groups (*p* < 0.05). There was a significant difference in carbohydrate and whole grain intakes between classification of adherence HNFI score in case subjects (*p* < 0.05).

**Table 1:** Comparison of energy-adjusted dietary intakes in Nordic diet adherence between groups

Variable	Nordic diet adherence in case group (*n* = 120)			Nordic diet adherence in control group (*n* = 120)			P-value ^#^
	Low	Medium	High	Low	Medium	High	
†Energy and dietary macronutrient intake (g/day)							
Energy	2973.18 ± 1097.11	2818.40 ± 1040.73	2569.72 ± 859.49	2061.59 ± 882.10	2245.28 ± 770.43	1716.94 ± 521.43*	**0.036**
Carbohydrate	186.91 ± 113.94	232.79 ± 57.70	240.63 ± 79.81*	255.85 ± 79.03	265.32 ± 76.24	256.30 ± 42.78	0.180
Protein	83.48 ± 32.74	72.86 ± 19.25	77.77 ± 20.87	75.47 ± 16.42	83.92 ± 15.45	80.51 ± 9.27*	0.576
Fat	117.62 ± 47.40	107.84 ± 23.05	103.10 ± 30.72	97.03 ± 28.58	89.87 ± 28.79	94.61 ± 17.06	0.199
†**Components of Nordic (g/day)**							
Whole grains	52.86 (10.76-101.72)	83.68 (27.01–167.50)	97.22 (53.97-167.29)*	105.48 (59.67-184.65)	174.06 (60.86-330.13)	142.71 (115.53-192.07)	0.640
Legumes	7.8 (3.04–12.30)	9.39 (2.89–22.29)	14.17 (11.38–30.73)**	9.55 (4.18–16.19)	13.52 (6.96–20.51)	19.33(13.13–28.71)**	0.419
Fruits	3294.32 ± 373.96	3550.99 ± 399.64	3661.58 ± 414.26**	3348.42 ± 727.92	3370.99 ± 337.17	3427.69 ± 179.10**	0.168
cabbages and vegetables	78.60 (42.79-145.21)	117.13 (57.59-220.12)	169.80 (108.90-236.76)**	67.96 (42.93-101.28)	102.13 (51.54-170.98)	140.35 (117.34-181.73)**	0.290
Root vegetables	25.27 (16.77–51.32	35.04 (18.36–47.38)	46.33 (29.61–65.64)	21.23 (12.16–30.18)	23.34 (12.29–41.92)	36.95 (27.86–54.84)**	0.562
Fish	2.33 (0.28–5.44)	5.34 (2.53–8.41)	8.00 (4.83–10.20)**	4.19 (2.28–6.28)	5.97 (4.05–7.70)	6.33 (2.51–8.05)**	0.930

Obtained from Multivariate analysis of variance (MANOVA) test

Data presented as Median (IQR) or Mean ± SD

†The parameters are adjusted based on the energy intake

*p < 0.05 and **p < 0.001 within classification of Nordic diet adherence

# p-value for differences between case and control group

**Fig. 2:**
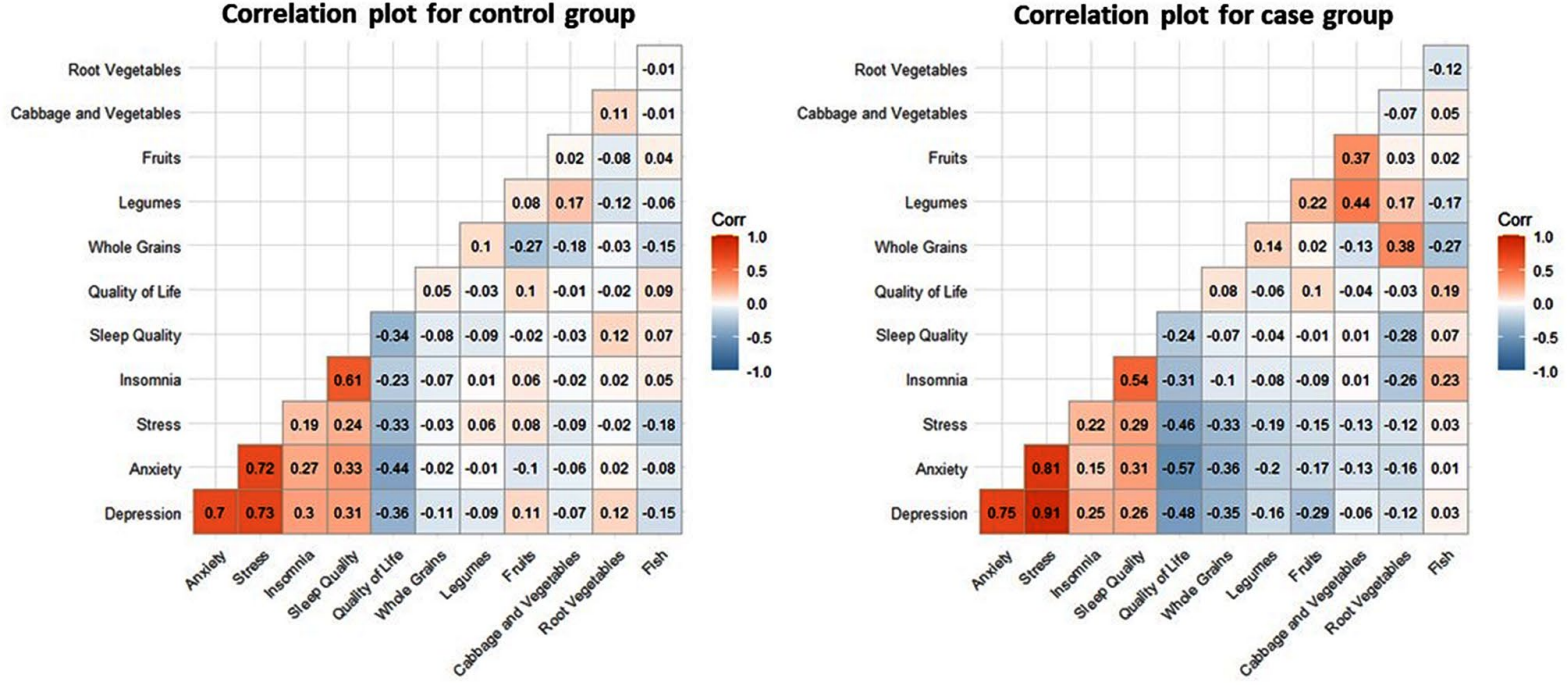
Correlation between components of Nordic diet and psychological tests in case and control groups

Heat map (Fig. 2) demonstrates that there was a significant opposite association between total anxiety, stress, and depression scores and the consumption of whole grains (*r* = − 0.35; *P* < 0.05, *r* = − 0.36; *P* < 0.05, *r* = − 0.33; *P* < 0.05 respectively). Furthermore, there was a significant opposite relation between depression and fruit intake (*r* = -0.29; *P* < 0.05). A significant negative association was observed between insomnia and sleep quality and the consumption of root vegetables (*r* = − 0.26; *P* < 0.05, *r* = − 0.28; *P* < 0.05, respectively).

Multinomial logistic regression analyses were applied to evaluate the relationship between psychological function and the Nordic diet in crude and adjusted models. As observed in Table 2, the odds ratio was adjusted for gender, age, educational levels, and energy intake in the adjusted model. High adherence to the ND was significantly related to anxiety, stress, and depression in the adjusted model (OR = 0.759, 95% CI 0.602–0.956, P-value = 0.019; OR = 0.719, 95% CI 0.563–0.918, p-value = 0.008; OR = 0.755, 95% CI 0.609–0.934, P-value = 0.010, respectively) only in the case group.

**Table 2:** Multiple-adjusted odds ratio (OR) and 95% confidence intervals (CI) for OR between classification of adherence the Nordic diet

	Nordic diet adherence in case group (*n* = 120)			Nordic diet adherence in control group (*n* = 120)		
	Low	Medium	High	Low	Medium	High
Anxiety						
Crude	Ref.	0.986 (0.862–1.128)	0.839 (0.723–0.973)	Ref.	0.988 (0.921–1.059)	0.980 (0.916–1.050)
Adjusted model	Ref.	0.997 (0.838–1.185)	0.719 (0.563–0.918) *	Ref.	1.002 (0.927–1.083)	0.972 (0.898–1.053)
**Stress**						
Crude	Ref.	1.006 (0.902–1.123)	0.849 (0.742–0.972)	Ref.	0.984 (0.940–1.030)	0.962 (0.919–1.007)
Adjusted model	Ref.	1.000 (0.863–1.158)	0.755 (0.609–0.934) **	Ref.	0.992 (0.941–1.045)	0.950 (0.901–1.002)
**Depression**						
Crude	Ref.	0.994 (0.884–1.117)	0.985 (0.734–0.994)	Ref.	0.993 (0.943–1.047)	0.044 (0.961–1.045)
Adjusted model	Ref.	0.979 (0.841–1.139)	0.759 (0.602–0.956) *	Ref.	1.005 (0.950–1.063)	0.982 (0.927–1.041)
**Sleep Quality**						
Crude	Ref.	1.063 (0.892–1.266)	0.964 (0.815–1.139)	Ref.	1.024 (0.949–1.105)	1.017 (0.945–1.095)
Adjusted model	Ref.	1.130 (0.850–1.501)	0.869 (0.634–1.192)	Ref.	1.045 (0.958–1.140)	1.049 (0.965–1.139)
**Insomnia**						
Crude	Ref.	1.134 (1.005–1.279)	1.064 (0.943-1.200)	Ref.	0.956 (0.872–1.048)	1.000 (0.918–1.090)
Adjusted model	Ref.	1.109 (0.953–1.289)	1.087 (0.915–1.291)	Ref.	0.981 (0.885–1.087)	1.076 (0.976–1.185)
**Quality of Life**						
Crude	Ref.	0.988 (0.956–1.021)	1.029 (0.999–1.059)	Ref.	1.015 (0.971–1.062)	0.995 (0.954–1.038)
Adjusted model	Ref.	0.969 (0.921–1.020)	1.056 (1.008–1.106)	Ref.	1.005 (0.955–1.057)	0.990 (0.942–1.041)

Obtained from multinomial logistic regression based on adherence the Nordic diet

Model 1: Adjusted for age, gender, education stage and energy intake

*p < 0.05

**p < 0.01

## Discussion

This case-control study evaluated the relationship between adherence to the ND and psychological role in 240 adults aged ≥ 30 years old who were healthy and recovered from COVID-19. In this study, we found that more adherence to ND was related to lower odds of anxiety, stress, and depression in recovered COVID-19 patients. Regarding components of the Nordic style, only in the case group, we found a significant opposite correlation between total anxiety, stress, and depression scores and the consumption of whole grains. Also, there was a significant opposite relation between depression and fruit consumption in this group.

The relationship between dietary patterns and psychological health has been considered an important issue [25]. Multiple studies recommended consuming food sources of vitamins and fibre during COVID-19, that are rich in the Nordic diet [26–28]. Choosing food like fruits, vegetables, and whole grains which are rich in fibre, antioxidant, and anti-inflammatory constituents might be important in COVID-19 [29]. Brown et al. found that a diet containing mostly whole grains, vegetables, and fruits with low amounts of foods with animal sources decreased the severity of COVID-19 [30]. Another study showed that increasing the intake of fruits, vegetables, and whole grains and decreasing the consumption of red meat, processed meat, sweets, refined cereals, fried food, and sugary drinks have antidepressant effects [31]. In line with our study, a randomized controlled trial performed in 2021 showed that a healthy Nordic diet improves depressive symptoms [13]. Also, a cross-sectional study with 181 subjects, aged between 18 and 25 years old, showed that adherence to a Nordic diet with a high intake of fruits and vegetables reduces stress and anxiety scores [11]. A plant-based diet rich in fibre, resistant starch, and carbohydrates appears to be advantageous because it fills the host’s intestinal with beneficial microbes that have health benefits for COVID-19 patients [29]. Enhancing diet quality improved mood. Dietary patterns rich in omega-3 and fibre may be related to decreased symptoms of anxiety, stress, and depression [32].

People suffer from mental problems after contracting COVID-19 due to the fear of losing people and social rejection. A dietary pattern rich in vegetables and fruits plays a role in improving mental distress [33]. In this study, we revealed that more adherence to ND was associated with less odds of anxiety, stress, and depression score through recovered COVID-19 patients. Our results were in line with the findings of prior studies [11, 13]. We concluded that depression scores were inversely associated with the consumption of fruit. Also, root vegetable consumption was correlated with insomnia and sleep quality among recovered COVID-19 patients. Root vegetable and Fruit intake improve life satisfaction and mental health. A meta-analysis consisting of 446,551 subjects, revealed that vegetable and fruit intake may play an essential function in reducing the depression risk [34]. Some studies estimated that the consumption of fruits can negatively affect mental health [35, 36]. Liu et al. in their meta-analysis indicated that fruit intake lowered depression and anxiety symptoms [37]. Various possible mechanisms could link fruit and vegetable intake with psychological symptoms. Oxidative stress has negative effects on mental health. A large number of antioxidants in vegetables and fruits, such as beta-carotene, folic acid, vitamin E, and vitamin C reduce the harmful oxidative stress effects on mental well-being and improve depression [38]. Fruits and vegetables are rich in different minerals and vitamins like folate. Folate and vitamin B12 deficiency increase the levels of homocysteine and the risk of depression [39]. Also, magnesium deficiency may increase inflammatory factors like C-reactive protein which helps the development of depression [40].

A healthy diet and lifestyle could affect symptoms of mood disorder in recovered COVID-19 patients [5, 41]. Inflammation caused by COVID-19 can affect neurological mechanisms, so having a healthy diet should be prioritized to prevent long-term neurological symptoms from COVID-19 [11]. Therefore, consumption of fibre and whole grains is recommended [42]. Our results revealed a significant relationship between whole grains anxiety, stress, and depression which confirms previous studies [43–45]. A cohort study found that regular consumption of whole grains, fruits, and vegetables is inversely related to anxiety and depression risk in elderly persons [6]. Mohammadi et al. in their randomized clinical trial study recognized a positive association between stress and anxiety and whole grains [44]. A previous study revealed that a greater intake of non-refined grains concluded to decrease depression and anxiety severeness [45]. A dietary pattern identified by high whole grain consumption was significantly connected with decreased depression risk [46]. In contrast, high consumption of refined grains was related to more depression risk [47]. Nutritional factors also have a direct and potent effect on neurophysiology [48, 49]. Berk et al. recognized inflammation as a mediating pathway for the development of depression [50]. Evidence suggests that frequent consumption of magnesium-rich foods may improve COVID-induced inflammation. A healthy diet provides sources of magnesium. For example, whole grains are identified as one of the best sources of food due to their magnesium content [51–53]. Also, a previous study revealed that a dietary pattern with higher intake of whole grains, fruits, and vegetables reduces inflammation by decreasing IL-6 and CRP in plasma [3]. Whole grains are a rich source of B vitamins like Thiamine, nicotinic acid, pyridoxine, and pantothenic acid but they are not rich in folates unless fortified with folic acid. These vitamins can positively affect mental health [54]. For instance, folate and pyridoxine deficiency are effective in mental health due to their function in the synthesis of neurotransmitters for example serotonin, as well as their coenzyme role in one-carbon metabolism pathways [55].

## Conclusion

Our study suggests that adherence to the ND may reduce anxiety, stress, and depression in patients recovered from COVID-19. A dietary pattern rich in fruit and whole grains might be beneficial in treating depressive symptoms in patients who have recovered from COVID-19. Additional large-scale longitudinal studies are essential to substantiate.

**Tables**.

## Data Availability

The datasets collected and/or analyzed during the present study are not publicly accessible due to ethical concerns but the corresponding author may provide datasets upon request.

## Abbreviations

ND: Nordic diet
FFQ: food frequency questionnaire
DASS: Depression anxiety stress scales
PSQI: Pittsburgh Sleep Quality Index
ISI: Insomnia Severity Index
SF-36: Short-Form Health Survey
COVID-19: Coronavirus disease 2019
SD: standard deviation
BMI: body mass index
HNFI: Healthy Nordic Food Index
IQR: interquartile range
MANOVA: Multivariate Analysis of Variance
SPSS: Statistical package for social sciences
